# Protein Consumption and Cognitive Health in Aging: Associations with Sarcopenia and Dietary Options, a Narrative Review

**DOI:** 10.3390/nu18132148

**Published:** 2026-07-02

**Authors:** David McCarthy, Aloys Berg

**Affiliations:** 1 School of Human Sciences, London Metropolitan University, London N7 8DB, UK; 2 Faculty of Medicine, University of Freiburg, 79117 Freiburg, Germany; berg.aloys@web.de

**Keywords:** protein, requirements, cognitive health, dementia, sarcopenia, elderly, soy, dairy, functional foods

## Abstract

The growing aging population is a primary driver of chronic, long-term health conditions. The rising prevalence of cognitive decline in older populations is a pressing public health issue due to its impact on health and social care and its emotional toll on family members. A lesser-known condition is sarcopenia—the age-related debilitating loss of skeletal muscle mass and function which leads to weakness and loss of mobility, quality of life and social independence. Neither health condition has a clear pharmacological treatment pathway. Diet and nutrition have therefore received the most attention for disease prevention. This review evaluates the research on the association between sarcopenia and cognitive decline and how both conditions may be linked to protein intake. While findings can be inconclusive or contradictory, a higher consumption of protein may protect against declines in physical and cognitive health, either acting separately or synergistically with exercise. The evidence supports the recommendation for a daily intake of protein higher than the current guideline of 0.8 g/kg/d for older people. Evidence suggests that healthy dietary patterns such as the Mediterranean diet appear to positively influence cognitive health in older people. Furthermore, the impact of specific high-protein foods, including egg, soy and dairy foods, on cognitive health has been reviewed, again with a suggestion that their consumption may mitigate against cognitive decline. Functional foods aimed at the aging population who wish to increase their protein intake and avert or delay the onset of these health conditions could play an important role in preventive nutrition, especially if they are formulated around the protein-rich foods which appear to positively impact cognitive health.

## 1. Introduction

The ever-growing aging population across the globe continues to be one of the most important public health challenges facing the twenty-first century. Whilst the advance in human longevity has been achieved though improvements in nutrition, infection control, socio-economic conditions and healthcare during the last century, aging also brings an increasing burden of non-communicable diseases including cardiometabolic disease and cancers [1]. These clinical conditions account for most of the ill health in older age and naturally have received the greatest research attention and healthcare input. Alongside these major conditions, there are a group of age-related degenerative diseases which include osteoporosis, sarcopenia and functional cognitive decline. Collectively, the impact upon ever-decreasing healthcare resources on managing these conditions is unsustainable in the long term and greater focus needs to be directed towards preventing, delaying or slowing the progress of these diseases. Diet and lifestyle approaches clearly fit into this strategy. Additionally, if more evidence-based, cost-effective treatments such as dietary interventions could be exploited for the management of cases of existing diseases, this should also be prioritized. Compared with cardiometabolic disease and cancers, understanding of the dietary associations with sarcopenia and cognitive decline are less advanced, but nevertheless offer great opportunities for potential preventative and therapeutic approaches. In this article, the current literature on the inter-relationship between sarcopenia, cognitive decline and role of diet, with a particular focus on protein, will be reviewed, with the aim to identify potential dietary options in their prevention, delay and therapeutic management. The approach is to focus, as far as possible, on original studies—cross-sectional, longitudinal or intervention, and complemented, where appropriate, with focused reviews.

## 2. Sarcopenia and Cognitive Decline: Definitions, Prevalences and Inter-Relationships

Sarcopenia refers to the age-related loss of skeletal muscle mass and functionality. Remaining an under-recognized degenerative disease, it can result in poor muscle strength, physical weakness, and mobility problems. This decline in physical health can lead to reduced walking capacity, loss of functional and social independence, frailty and poor overall quality of life. Figures cited by Petemann-Rocha et al. (2022) using data from the European Working Group on Sarcopenia in Older People estimate that sarcopenia prevalence varied between 10 and 27%, dependent upon the choice of classifications and cut-off points [2]. Age groups and sex also influenced the estimates, with older age demonstrating increasing impact and severity. Currently, there is no approved pharmacotherapy for the condition. Given the physical impact of sarcopenia on individuals and the subsequent toll on family support, the economic burden of related health and social care expenditures is substantial. A detailed review of the status of the pathophysiology and emerging therapeutic options for sarcopenia is provided in Araujo et al. (2025) [3].

Cognitive decline also presents as an illness associated with advancing age, with a continuum of stages progressing from mild to moderate to severe cognitive decline or dementia. Moreover, different forms of dementia exist including, amongst others, Alzheimer’s disease (AD) and vascular dementia. Its main characteristics include impaired memory, decline in communication ability and difficulties performing everyday tasks. Cognitive decline is also one of the most pressing clinical and public health issues society faces in the 21st century, especially with the rising numbers of the population living to old age [4]. Dementia places a huge economic, social and emotional burden on healthcare providers and relatives and great distress to those who suffer from the condition. Currently, pharmacological treatment options are limited in range and effectiveness, and beyond routine healthy lifestyle advice and modification, more needs to be understood about how this aging-related condition can be prevented or delayed. However, there is emerging evidence which identifies how tackling specific risk factors can reduce the risk of developing dementia [5]. Additionally, for both aging-related conditions, the roles of diet and specific nutrients have been extensively studied, in relation to risk for developing these illnesses as well as their role in prevention, delay and treatment.

## 3. Inter-Relationships Between Measures of Sarcopenia and Decline in Cognitive Health

There is an accumulation of evidence to suggest that physical decline (in the form of sarcopenia) is independently associated with cognitive impairment in older age. However, this appears to be a complex relationship, with some earlier studies generating contradictory observations, making arriving at a definitive conclusion difficult. A common problem of studies on sarcopenia–cognitive decline associations include the definition and choice of measures of sarcopenia (such as DXA- or BIA-derived skeletal muscle mass, handgrip strength and gait speed) and the types of tools for assessing cognitive function. Additionally, most of these early studies tended to be observational in study design, thus limiting the interpretation of the findings. A 2002 cross-sectional study on over 7000 community-dwelling women aged over 75 years found that low muscle mass was associated with cognitive impairment. However, despite a suggestion that there could be common mechanisms, it could not predict the causal variable [6]. Two similarly designed cross-sectional studies in different population groups (Chinese men and women aged > 65 years and French women aged over 75 years) failed to show any significant associations between DXA-derived measures of lean/muscle mass and cognitive impairment (measured by a population-specific-based questionnaire/assessment tool). Nevertheless, functional-based measures of handgrip strength and gait speed were associated with cognitive impairment, suggesting that sarcopenia did not mediate the association between physical performance and cognitive impairment [7,8]. Over time, the scientific literature in this area has expanded into more sophisticated study designs, revealing further understanding. One of the first cohort studies examined non-demented community-dwelling older adults at baseline with a mean follow-up of 5.6 years. They found that those with more severe measures of sarcopenia at baseline had an increased risk of incident AD, incident mild cognitive impairment and a faster rate of cognitive decline [9]. Again, it was the muscle functional component rather than muscle mass that was the main driver of the association.

In more recent years, a further number of studies, varying in study design (prospective, case–control and observational), assessment tools, population group and sample size have been conducted to potentially corroborate earlier studies or to extend understanding of the association between measures of sarcopenia and cognitive impairment. Overall, the findings have tended to support the associations and begin to question whether management of sarcopenia could be an effective strategy to prevent cognitive decline, and if measures of sarcopenia could be used to predict mortality in those population groups [10,11,12]. These conclusions come with a caveat that a number of study limitations were identified, including sample size and cross-sectional nature of some aspects of the studies. A major advancement into the causal relationship between sarcopenia and cognitive decline utilized Mendelian randomization analysis on a large prospective cohort. Concurrent studies utilizing the UK Biobank data indicated that sarcopenic traits (muscle mass, handgrip and walking speed) were positively associated with cognitive performance and that sarcopenia is a potential causal risk factor for cognitive impairment. Furthermore, these studies also suggested that monitoring muscle health of older individuals and preventing or delaying the progression of sarcopenia could be a means of potentially slowing cognitive decline [13,14]. Likewise, another longitudinal study, this time based on a Chinese population (*n* = 4376), demonstrated that a diagnosis of sarcopenia was significantly associated with a decline in three cognitive domains (temporal orientation, memory function and global cognition), particularly in those with accelerated biological aging. The authors recommended that sarcopenia management should be integrated with strategies targeting accelerated biological aging to mitigate cognitive decline in older population groups [15]. Taking further this concept of monitoring muscle in older individuals, in a recent study utilizing longitudinal data in a Chinese population group, heterogeneous trajectories of appendicular skeletal muscle mass (ASM) changes were analyzed in relation to cognitive impairment. In summary, compared with those defined as a high slow decline in ASM, the moderate stable group and the low rapid decline group had significantly higher risk of cognitive impairment. The authors suggested the monitoring of ASM to facilitate early detection of populations at high risk of cognitive impairment [16]. This recommendation has been further supported by recent findings which showed that higher baseline measures of physical (muscular) function (including chair stand performance and handgrip strength) in elderly participants were significantly associated with more favorable 2-year trajectories in global cognition, memory, and executive function (*p* < 0.05). This was particularly true for memory. The authors concluded that having stronger muscles is linked to slower cognitive decline, even before its onset is observed. Additionally, better performance of muscular function is protective against cognitive impairment in aging, and intervening upon muscle health positively impacts brain health in aging [17].

Overall, the consensus of findings, which were derived predominantly from longitudinal studies, tended to agree that maintaining muscular health in middle-to-older age (whether based on mass or function) is key to preventing, delaying or minimizing cognitive decline. Support for muscle health would include physical activity and exercise strategies as well as dietary approaches. In relation to the latter, focus could be on whole-dietary patterns or specifically targeted nutrients, especially dietary protein. These approaches could also impact upon cognitive health directly, independent of the role of muscular health. 

## 4. Sarcopenia and Dietary Protein

The past 20 years has generated a wealth of research evidence into the association of dietary protein intake and sarcopenia. Studies have explored this observationally as well as prospectively and through interventions to address the following general research questions:

1. Whether (inadequate) protein intake could be a risk factor for developing the condition.
2. If increasing protein intake could delay, prevent or reverse the progression of the condition.
3. Whether certain protein-dense foods are better at supporting muscle health, and what their nutritional characteristics are.

The volume of research conducted in this area so far has been extensively reviewed elsewhere [18,19,20]. To limit unnecessary replication, a brief overview is provided, permitting greater focus on the most recent pertinent findings. However, context must first be provided in relation to dietary protein and requirements and the impact of aging. Dietary proteins vary in their effectiveness at supporting protein synthesis, reflected in the protein quality. Beyond digestibility, quality is dependent upon the essential (indispensable) amino acid (EAA) content and their ratio to each other. Measures including limiting amino acid (LAA) and amino acid score (AAS) are indicators of protein quality, where the pattern of amino acids in a food is compared against a reference or “ideal” pattern. Currently, the Digestible Indispensable Amino Acid Score (DIAAS) is the preferred method of assessing dietary protein quality, being a more accurate measure of amino acid absorption in the terminal small intestine. Animal proteins including eggs, meat, fish and dairy have a higher quality compared with plant sources including legumes, grains, nuts and seeds, based on the amino acid characteristics indicated above. Protein quality can be enhanced by a combination of protein-rich foods which compensate for each other’s LAA, known as complementary proteins. An example would be a combination of dairy with legumes.

Guidelines from the World Health Organization (WHO/FAO/UNU, 2002) and the US Recommended Dietary Allowances give reference intake values for protein of around 0.8 g/kg/d for adults of all ages—a figure deemed to meet the needs of 98% of the population [21,22]. The US report concluded at that time that there was no evidence of increased protein requirements in older age above the safe level of intake, despite the importance of adequate protein consumption to support skeletal muscle mass. However, this assertion has been questioned for the elderly population, particularly for maintaining their muscle mass. Older age is accompanied by a reduced muscle protein synthesis following consumption of a protein-rich meal (compared with healthy younger adults) and there may be a threshold below which muscle protein synthesis cannot proceed optimally in older individuals [23]. This phenomenon of older age has been termed “anabolic resistance” and is thought to be a principal determinant of age-related loss of skeletal muscle mass [24]. Furthermore, this resistance appears to be exacerbated by physical inactivity. At a molecular level, the essential branched-chain amino acid (BCAA) leucine specifically acts as the most potent trigger for muscle protein synthesis following a protein-rich meal. The rise in blood levels of leucine following a leucine-rich meal acts as the initiator of muscle protein synthesis [25]. As further evidence of the importance of leucine in the preservation of muscle, consumption of a leucine supplement partially protects both muscle mass and function during bed rest in middle-aged adults [26].

Cross-sectional comparative studies of sarcopenic and non-sarcopenic older adults cited by Coelho-Junior et al. 2022 [18] overall found that protein consumption was significantly lower in the sarcopenic groups. Furthermore, replacing the animal-derived sources of protein with an equal amount of plant-derived protein resulted in a lower risk for sarcopenia. In support of these observations is a recent cross-sectional study of elderly Japanese women. Muscle mass, strength, muscle-specific strength and physical performance were assessed in relation to a protein-rich food intake frequency score. Those with a higher intake of the protein-rich diet had better muscle mass, strength and physical performance but not specific muscle strength. They concluded the importance of protein intake in preventing sarcopenia [27]. Supporting evidence has come from a 2024 community screening study for sarcopenia in Australian adults aged >65 years. It demonstrated that a “low” usual intake of protein (<1 g/kg/d) together with low levels of physical activity can identify those with lower percentage muscle mass, higher fat mass, poorer strength and physical function and hence sarcopenia [28]. In contrast, a UK cross-sectional study on healthy twins aged 60 years+ showed that a high intake of protein (>1.3 g/kg/d) was associated with increased sarcopenia. Furthermore, no difference in protein intake was observed when comparing those either with or without low muscle strength. In this study, the majority of protein intakes were animal derived [29]. The authors suggested that baseline protein intake should be considered when advising older adults on dietary intake and that longitudinal studies in cohorts with larger numbers of individuals with sarcopenia should be undertaken to further assess this association. Taken together, because these observational findings are subject to the limitations of being unable to establish causation as well as potential bias and confounding, they demonstrate a complex and incomplete understanding of the dietary protein–sarcopenia inter-relationship. Longitudinal and intervention studies may assist in unravelling this complexity.

Longitudinal studies on protein consumption and sarcopenia risk and development are fewer in number. One of the first was conducted on a UK population aged 85 years+ with the aim to investigate typically British dietary patterns on sarcopenia risk and determine if the level of protein intake influenced the relationship. A key finding was that a dietary pattern high in traditionally British foods (butter, red meat, gravy and potato) where the intake of protein was considered a “good level” (>1 g/kg/d) was associated with a greater risk of sarcopenia [30]. A study conducted on an Australian cohort aged 60 years+ (AusDiab) examined the relationship between protein consumption and health-related quality of life outcomes (HRQoL) over a twelve-year period. HRQoL included a physical component score and mental component score. The findings demonstrated that total dietary protein intake was not associated with changes in HRQoL. However, higher intakes of animal, red meat and processed animal protein were associated with detrimental changes in either Physical Component Summary (PCS) and/or Mental Component Summary (MCS) scores. The authors suggested that protein sources should be considered when advising older adults on protein consumption [31]. A prospective cohort study on Chinese community dwelling adult aged 40–75 years examined protein intake in relation to appendicular lean mass (ALM) and its index (ALMI). Over a 3.2-year follow-up, total protein intake was shown to preserve ALM and ALMI in a dose-dependent manner in both sexes. In women, the risk for sarcopenia was reduced by 35–50% in the highest intake of protein, regardless of whether the protein was of animal or plant origin [32]. Given the nature of the study design, the authors were able to confidently conclude that an adequate protein intake was more effective at preventing loss of muscle and sarcopenia, rather than protein sources. These observations contrasted with those longitudinal findings discussed in this section. A case–control study explored the association between a plant-based diet and sarcopenia risk. Whilst being small-scale and not specifically focusing on the protein aspects of the diet, findings revealed a negative association between a plant-based diet and the odds of sarcopenia. Caution should be exercised, however, as statistically underpowered studies may be unable to detect true associations. Further research, involving better designed methodologies, could reveal whether the plant protein sources could account, in part, for this protective effect, together with the range of micronutrients supplied in this type of diet [33].

Randomized controlled trials (RCTs) are considered the gold standard for evidencing causal relationships due to their low inherent bias when conducted appropriately. Consequently, this experimental approach has been exploited in intervention studies of protein intake and muscle health in older age. Unsurprisingly, these studies have varied in study design, including population group, sample size, age, health status, baseline protein intake, duration of intervention, source/type of protein, with or without an exercise component and outcome measurements including muscle mass, physical performance or sarcopenia. It is beyond the scope of this review to provide a comprehensive analysis of the findings. However, a consensus suggests that providing additional protein (irrespective of source, type, or format of provision) has either a neutral or clinically meaningful positive effect on muscle health/sarcopenia risk, albeit ranging in impact. This is evidenced in a selection of recent trials [34,35,36,37,38,39,40,41]. In these studies, protein supplementation originated from whey protein, soy protein isolate or unnamed sources. Given the known role of leucine in triggering muscle protein synthesis, it is to be expected that RCTs have been conducted to evaluate the impact of supplements of BCAAs with or without an exercise component on outcomes of muscle mass and function in the older population. Generally, findings have varied with respect to outcome measure but ranged from zero impact to maintenance of lean body mass [42,43,44,45]. Caution should be exercised when interpreting these findings due to variations in sample size, duration of intervention, participant adherence to the protocol and quality of body composition assessment techniques. Overall, the findings suggest that additional research is warranted to clarify the impact of BCAA supplementation on sarcopenia risk and muscle mass and function in the elderly.

Whilst the focus here has been exclusively on the protein aspect of diet and sarcopenia risk, a clear indicator across all the research is the central role of physical activity, especially strength-based forms of exercise, on skeletal muscle preservation. This is key to any strategy to prevent, delay or reverse age-related decline in muscle health and is addressed across a range of focused reviews [46,47,48].

Drawing upon the accumulated data on habitual protein intakes in older people, and the suggestion that the more the intake of protein falls below the reference figure of 0.8 g/kg/d the greater the risk of loss of skeletal muscle mass, it has been suggested that protein requirements for the elderly may actually be greater than this daily reference amount [49]. Evidence suggests a protective role for extra protein in older adults to preserve muscle mass and to prevent frailty, leading to a proposition of recommending a higher reference value for older people [50]. Thus, it has been reported that for maintaining good health in aging populations, a protein intake of at least 1.0–1.2 g/kg/d is needed, leading to 1.2–1.5 g/kg/d for those with acute or chronic conditions. In individuals with protein-related malnutrition including sarcopenia, a higher daily intake of protein of around 1.0–1.5 g/kg/day has been suggested [51]. Whilst the WHO and US protein guidelines continue to maintain the 0.8 g/kg/d figure for the elderly, several other countries, including Germany, Austria and Switzerland (over 65 years), the Nordic countries (61–74 years) and Australia/New Zealand (>70 years), have adopted a higher protein reference value [52,53,54].

Considering the recognized association between sarcopenia and risk of cognitive health decline, it is noteworthy that very few of the studies on protein intake and muscle health in the elderly have incorporated measures of cognitive function in the study design. Additionally, while there is a clear physiological basis for examining protein intake in relation to skeletal muscle health in the elderly, no similar physiological rationale could be claimed for cognitive health. Dietary protein, however, is the source of amino acid precursors to neurotransmitters and neuropeptides. It is critical to CNS structural architecture and may also influence neuronal function, intracellular signaling, cerebral blood flow (CBF) and neuroinflammation. Consequently, a body of research has explored the association between diet on cognitive health in the elderly, some of which has focus on the protein aspect.

## 5. Protein Consumption and Cognitive Health in the Elderly

Data on protein consumption and cognitive health has been gained from a variety of research methodologies including observational, longitudinal and interventions. Consequently, the hierarchy of evidence principle defines the quality of the findings, and cognizance of this must therefore be paid when interpreting the outcomes of the various studies and how they rank in their relative strengths and weaknesses. The population groups examined also vary including memory and cognition studies in healthy adults and older individuals, to those with varying degrees of cognitive impairment. One of the earliest studies was a population-based prospective cohort which explored the relative intake of macronutrients compared to risk for mild cognitive impairment or dementia in a sample of 937 participants with a median age of 79.5 years and followed over a median 3.7 years [55]. Relative risk was lower in those who had a higher % energy intake from protein or fat. Dietary data was obtained from a food-frequency questionnaire which is considered satisfactory for ranking individuals with respect to food and nutrient intake. A 2018 cross-sectional study also examined macronutrient intake in relation to cognition [56]. In this Chinese population aged <65 years, reverse findings were observed to those above, with individuals consuming a greater proportion of their energy from protein and fat having a higher association with mild cognitive impairment. Again, given the nature of the study design, no cause and effect can be inferred. However, it was interesting to note that as a population undergoing a nutrition transition from a lower to a higher intake of protein, this may have played a role in these observations. The authors stated, however, that an optimal intake of protein for cognitive health remained unknown.

A 2020 cross-sectional study using The National Health and Nutrition Examination Survey (NHANES) 2011–2014 dataset demonstrated a positive association between total protein intake, as well as specific sources of protein including legumes, eggs, meat and total animal protein, and cognitive function in individuals aged 60 years+. However, an adverse association was observed with milk and milk products [57]. The authors highlighted several discrepancies between their findings and those of others at the time. This suggests a need for more higher-quality designed studies to help disentangle the complex relationship between protein consumption and cognitive health.

A 2022 study has advanced the understanding of long-term dietary protein intake and subjective cognitive decline drawing upon longitudinal data over a 20-year period, from two large US cohort studies—the (female) Nurses’ Health Study (121,701 participants, NHS) and the (male) Health Professionals Follow-up Study (51,529 participants, HPFS) [58]. Dietary intake was assessed using a semi-quantitative food frequency questionnaire and subjective cognitive decline (SCD) using a questionnaire-based methodology. In summary, higher total, animal and plant protein intakes, adjusted for total energy intake were significantly associated with lowers odds of SCD in both males and females. In addition, even lower odds of SCD were observed when animal protein was substituted for plant protein. A significant association with better subjective cognitive function in later life was observed, with the consumption of legumes/beans, fish and lean poultry. Furthermore, incremental substitution of energy from total protein for that from total carbohydrate was associated with lower odds of SCD; in this context, plant protein substitution had the strongest inverse association. The study was also able to advance knowledge of the contribution of the intake of individual amino acids to lower odds of SCD, with proline standing out in both cohorts. Certainly, the findings have important public health implications, and the authors recommended further studies to verify their findings.

A genetic risk for late-onset AD has been identified with the inheritance of alleles of the apolipoprotein E (APOE) gene. This gene codes for a 299 amino acid protein with the APOE-ε4 variant present in ~25% of the US population, with at-risk individuals having an earlier onset of the disease [59]. Studies have considered whether there may be an interaction between the APOE-ε4 allele carriage and protein intake on cognitive decline. A 2021 study using data from the Chinese Longitudinal Healthy Longevity Survey (CLHLS) demonstrated that participants carrying the APOE-ε4 allele and who had high total protein intake or fish protein intake showed significantly lower odds of cognitive decline, despite no overall association between the protein intake dietary diversity or the consumption of six protein-rich foods and cognitive function [60]. A later study examined the influence of the APOE-ε4 allele on the association of total protein intake with cognition specific to AD, and particularly the decline in episodic memory in older individuals without dementia. Using a more robust assessment of memory, the general observations were that a higher intake of protein was associated with better cognition and episodic memory in those without dementia, and the APOE-ε4 allele was an important moderator in the association. The authors concluded that cognitive health could benefit from dietary interventions that focused on protein content, especially in those with a genetic predisposition to AD [61].

Most recently, additional studies have added to the evidence base for an association between protein intake and cognitive health in older individuals. In particular, the dynamic pattern of protein intake over a 10-year period and cognitive function has been assessed, also drawing upon data from the CLHLS. Whilst an upward trend in the frequency of protein consumption in older individuals was observed, in contrast, cognitive function exhibited a downward trend. It was concluded that an increased frequency of protein consumption may protect cognitive function in older individuals, and sustaining a consistently high frequency of protein intake could be a significant factor in stabilizing cognitive function in older individuals [62]. Protein consumption has also been evaluated across three meals, breakfast, lunch and dinner, in relation to a range of cognitive tests in older US adults, including Hispanic adults, using NHANES data. Those in the highest quintile of dinner protein intake demonstrated a lower risk of low cognitive performance compared with those in the lowest quantile. This was observed to a lesser extent for lunch protein but essentially none for breakfast protein intake. Given the timing of the meals across the day, the authors raised the possibility of a circadian rhythm effect of protein intake on cognitive function [63].

In much the same way that research on protein intake and sarcopenia risk has tended not to incorporate assessment of cognitive health into the study design, the reverse is also largely true in the studies reviewed above. Thus, opportunities may have been missed to examine the inter-relationships between protein intake, sarcopenia risk and cognitive health. One study, however, has attempted to explore this three-way relationship using data from NHANES [64]. In a population of older adults, the interaction between handgrip strength, cognitive function and dietary protein was investigated cross-sectionally. Greater grip strength was associated with better cognitive performance, and a weak handgrip was associated with both cognitive impairment and a low intake of protein. A significant interaction effect of protein intake was observed. Given the limitations of a cross-sectional study design, the authors concluded that improving muscle strength and increasing protein intake may be effective strategies to mitigate decline in cognitive function in older individuals.

On balance, the literature provides an intriguing possibility that additional dietary protein could mitigate against cognitive decline in older age. However, caution should be exercised in interpreting the findings due to a likelihood of bias and confounding, which is inherent in some of the study designs. There are well-known limitations with the tools for assessing dietary intake, often relying upon recall, and, together with misreporting, introduces measurement error. Used often in the studies reported, a FFQ is better at ranking intakes of nutrients rather than providing quantitative data on nutrient intake. For the assessment of cognitive function, questionnaires are often employed, which, whilst usually validated, are still prone to response bias and misinterpretation. These are critical issues which must be acknowledged when judging the outcomes of studies. Given these known limitations, an opportunity remains to hypothesize on the nature of such a possible protective effect of dietary protein and to translate this into practical applications. Whether there is a direct effect of amino acids on brain health and cognitive function, or whether protein acts indirectly via anti-inflammatory or other properties such as via the gut microbiome are worthy of consideration.

Achieving an optimum intake of dietary protein therefore appears to be critical for the physical and cognitive wellbeing of older adults. This optimum intake may well be at the higher level as indicated in Section 4 above. Alongside this suggestion, questions remain regarding whether this optimum protein intake can be achieved via a general varied and healthy dietary pattern and whether there are specific protein foods which can benefit muscle and cognitive health in older age. The diversity of food proteins varies in critical properties such as quality, amino acid composition, digestibility and the presence of bioactive peptides and co-nutrients. Specific studies discussed above have been able to identify certain foods and food groups, such as legumes/beans, eggs, meat, fish and dairy, that may be beneficial or detrimental to cognitive health in older adults. Additionally, consumption of supplements, including amino acids, should be evaluated. Evidence for dietary strategies, including the impact of specific protein foods and amino acid supplements on cognitive/physical health in older adults, is now reviewed.

## 6. Whole-Dietary Patterns as Strategies for Cognitive and Physical Health in Older Adults

Specific dietary patterns are promoted for optimal health and longevity, particularly in relation to cardiometabolic health and the low risk for non-communicable diseases, including certain cancers. Thus, the Mediterranean diet (MD) is an exemplar in this context, defined by its emphasis on vegetables, fruit, legumes, wholegrain cereals, fish including oily fish, high ratio of mono to saturated fatty acids and lower proportions of dairy and red meat [65]. The EAT-Lancet diet is gaining recognition for its health-promoting properties and is characterized by its similarity to the MD but with limited intake of animal source foods and an emphasis on the intake of plant foods including nuts, seeds and berries [66]. The MIND (Mediterranean-DASH Intervention for Neurodegenerative Delay) diet is a hybrid of a typical MD and the DASH diets—the latter focusing on an increased intake of fruits and vegetable, wholegrain and low-fat dairy foods as well as reducing intake of salt to combat hypertension and to lower blood pressure [67]. All these diets have been evaluated in relation to cognitive health, with consideration given to the prevention, delay in onset, slowing of progression and management of the condition. A 2024 systematic review evaluated the adherence to an MD and risk of all types of dementia in populations aged 60 years+. Overall, an 11% reduction in risk was found for all dementias and a 27% reduction for AD only [68]. The authors acknowledged that caution should be exercised when interpreting their findings. However, given that dementia affects a relatively large number of older people, they suggested that even a small percentage reduction represents a significant number of people whose dementia could be prevented by adherence to the MD.

It must be recognized that there are several limitations to the MD. It is not necessarily culturally acceptable to large parts of the globe, as well as having issues with accessibility and affordability. Indeed, studies have researched the barriers and facilitators to adopting this type of diet in relation to cardiovascular health, with the aforementioned issues being highlighted as well as concerns around cooking skills, limited food knowledge and time commitment [69,70]. Furthermore, it would need to be adapted to match the food choices of vegetarians or vegans and can de-emphasize certain foods which may help boost cognitive health. To accommodate any higher recommendation for protein intake, it is likely that dietary modifications would have to be made to accentuate the content of protein-richer foods. Indeed, a modified, high-protein version of the MD has been devised, evaluated and found to be generally acceptable in cardiac rehabilitation patients. In this trial, a selection of protein-rich recipes incorporating foods included in the MD formed the basis of the high-protein element. However, the quantitative aspect of the daily protein intake of the study participants is unstated and a clear limitation of the trial [71]. A protein-enriched MD has been developed and evaluated in adults at risk of undernutrition and cognitive decline [72]. The diet aims to achieve a protein target of 1.5 g/kg/d though setting goals for dietary behaviors. Outcomes from the study are currently unpublished.

The EAT-Lancet diet has been evaluated in relation to cognitive health and function in a small number of population groups as well as in relation to sarcopenia risk. Studies (mainly longitudinal and intervention) have varied in their individual design, including the tools for assessment of cognitive function. Generally, a positive association between the EAT-Lancet diet and several measures of cognitive function was observed, together with a slowing of cognitive decline with age [73,74]. Additionally, an interplay between the diet and theAPOE-ε4 status has been identified, with a risk reducing effect found among non-carriers of the allele whereas no association was found within the APOE-ε4 carriers [75]. One observation of this dietary pattern is that a definitive figure for the protein level for a 2500 kcal/d intake is not apparent and would likely need to be modified to achieve close to a recommended protein target range of 1.0–1.5 g/kg/d. The EAT-Lancet diet has also shown that adherence to its dietary patten is associated with a lower risk of sarcopenia and sarcopenic obesity, mediated in part by inflammatory biomarkers [76].

Similar outcomes have been observed with the MIND diet, showing a substantial slowing of cognitive decline over a 4.7-year period in a US population with a mean age of 81.4 years [77]. A further study demonstrated a reduced risk of AD with adherence to the MIND diet over an average period of 4.5 years, suggesting such a high-quality diet can provide protection against dementia [78]. However, when evaluated in relation to genetic risk for dementia, no gene–MIND diet interaction was observed across three distinct population groups studied in relation to cognitive health, even though adherence to the MIND diet as well as having a lower genetic susceptibility were independently associated with a lower risk of dementia [79]. This positive impact of the MIND diet on dementia risk is supported though objective evidence from longitudinal brain imaging. A slower decline in gray matter volume and a slower increase in lateral ventricular volume were observed with greater adherence to the MIND diet over a period of around 12.3 years in middle-aged and older individuals [80]. This supports the suggestion that the MIND diet could be a strategy to delay structural brain aging and prolong brain health. However, it must be noted that not all studies have demonstrated positive outcomes, including a trial in cognitively unimpaired individuals with a family history of dementia [81]. No significant difference was observed in changes in cognition and brain Magnetic Resonance Imaging outcomes in those who followed the MIND diet and those who followed a control diet. Similar to the EAT-Lancet diet, the daily protein content of the MIND diet is unclear and is likely to be of a moderate level due to the focus on plant-based foods and a limited intake of red meat and cheese. This diet would have to be modified if it were to reach the higher protein intake levels of around 1.0–1.5 g/kg/d.

Recently, one of the first studies has been conducted to explore the interaction between dietary patterns, protein intake, sarcopenia and cognitive decline. The population was a sample of community-dwelling Hong Kong elders aged ≥ 65 years who formed part of the Mr OS and Ms OS prospective cohort, followed up over a 4-year period. Dietary patterns included the Diet Quality Index-International, DASH diet, MIND diet, Mediterranean Diet Score and the Dietary Inflammatory Index. The researchers found that dietary patterns and protein intake, particularly plant protein, were significantly and positively associated with cognitive outcomes, with these associations being more apparent in those with sarcopenia. Hand-grip strength and walking speed potentially mediated the associations. It was concluded that promotion of healthy eating patterns in older adults is a clear public health strategy which could help delay both cognitive and physical decline [82]. This is one of the first studies to describe a diet–muscle–cognition triad, a characterization which could be extended to a diet–protein–muscle–cognition tetrad, given the known and independent role of protein on cognitive health. In a further examination of this type of interaction, a 2026 study on English adults aged ≥ 65 years assessed associations between protein intake and two healthy eating patterns—a MD and the WHO Quality Diet Index—and measures of functional decline [83]. Findings showed that a higher intake of protein, especially when consumed at a level of 1.0 g/kg/d, was prospectively associated with lower risk of a range of measures of physical and functional decline and lower mortality risk. Furthermore, beneficial associations between protein intake and the healthy eating patterns were found, and this was particularly the case for animal protein. Overall, this study supported the evidence for beneficial properties of a higher protein intake, when consumed as part of a typical healthy eating pattern in the elderly, to support healthy aging and preserving physical function.

Whilst population-based studies such as those described above are valuable in assessing the impact of dietary patterns on health outcomes, they are also subject to limitations including diet adherence, self-reporting bias, and unaccounted confounding variables. Their strengths lie in sample population numbers and being more predictive of disease. Nevertheless, it should be reiterated that dietary patterns are not necessarily transposable between population groups where characteristic food behaviors and food availability may predominate.

The whole-diet approach in the elderly, however, is a rational strategy as it emphasizes the intake of a range of beneficial nutrients and bioactive compounds as well as limiting the intake of those considered less beneficial or detrimental to both cognitive and overall health. Hitherto, protein appears to be particularly beneficial, with conflicting evidence to date as to whether it is plant or animal protein (or neither) which offers a greater cognitive/physical health benefit. However, if the protein content of the diet is to be increased as the evidence and recommendations suggest, modifications to the whole-diet strategy would need to be made to accommodate this extra protein.

## 7. Impact of Selected Protein Foods and Amino Acids on Cognitive Health in Older Adults

Beyond the whole-dietary pattern, there is also potential for emphasizing the consumption of specific foods due to their characteristic composition and properties and possible benefit to cognitive and physical health. In this context, studies on egg consumption, soy protein and dairy in relation to cognitive health in older adults are reviewed.

### 7.1. Egg

With its unique nutritional profile, egg consumption could be important in relation to cognitive health and dementia risk. A highly digestible, protein-dense food with an amino acid profile as close to the “ideal” reference pattern as any food protein, chicken egg is rich in essential amino acids (EAAs) and a wide range of micronutrients and fatty acids. Egg is also the highest dietary source of choline—a micronutrient with a key role in brain function—as a precursor to the neurotransmitter acetylcholine and an integral component of cell membrane structure as phosphatidylcholine. Eggs could be considered an optimal dietary component to support cognitive health in older age, helping to delay the onset or slow the progression of dementia. If consumed as a regular supplement, it could help bring dietary protein intake closer to the recommended higher intake.

A suggestion that egg consumption could be associated with a reduced risk for dementia, and specifically for AD, was first explored in a Spanish dementia cohort of the European Prospective Investigation in Cancer and Nutrition. This observation was only found, however, in those who had a low adherence to an MD, and no association was found in those who had moderate and high adherence [84]. The authors speculated that a range of nutrients within egg could offer protection against cognitive decline, including protein, via a beneficial effect on inflammation and choline via its presence in phosphatidylcholine.

A 2024 prospective study followed 1024 older adults (mean age 81.38 years) over a period of 6.7 ± 4.8 years. Egg consumption was assessed with a semi-quantitative food frequency questionnaire. Weekly intakes of >1 egg/week and ≥2 eggs/week were associated with a decreased risk of AD. Furthermore, brain autopsies showed that the same levels of egg intake were associated with a lower risk of AD pathology. In addition, 39% of the total effect of egg intake was mediated by dietary choline [85]. A case–control study on a Chinese population examined frequency of egg consumption in relation to dementia. An inverse association between egg consumption and dementia was observed and concluded that a daily intake of egg could help reduce the risk of dementia [86]. A 2026 study explored the incidence of AD in the Adventist Health Study-2. With a sample of 39,498 participants and a mean follow-up period of 15.3 years, egg consumption was assessed using a Food Frequency Questionnaire (FFQ) and AD diagnosis determined from Medicare records. Again, a significant inverse relationship was found between egg consumption and AD risk, suggesting a neuroprotective property of the egg nutrient composition [87]. However, although inconsistent findings have also been reported [88], a 2026 meta-analysis concluded that consumption of 1 egg per day (50–60 g) is necessary for the maintenance of cognitive health in the elderly and as an effective food supplement to alleviate cognitive decline [89]. Taken together, the overall observations would suggest that regular egg consumption could benefit cognitive health in older individuals. Identifying potential mechanism(s) of action would strongly enhance any recommendations for cognitive health. However, despite the mainly positive evidence, it must be recognized that eggs do not form a part of all dietary patterns and can trigger allergic reactions in susceptible individuals who are advised to avoid eggs in their diet. Thus, alternative foods for cognitive health need to be considered when egg consumption is not an option.

### 7.2. Soy

Soy (soybean) is a globally consumed legume, being a staple across most parts of south and east Asia, but makes a minor contribution to a typical “Western” or MD. It is characteristically a protein-rich food with a high quality based on its the Digestible Indispensable Amino Acid Score (DIASS) due to the presence, availability and “complete” pattern of essential (indispensable) and conditionally indispensable amino acids [90]. Soy also contains isoflavones (genistein and daidzein), plant compounds with biological properties, and is also a source of biologically active peptides (lunasin) and soyasaponins [91]. Soy is consumed in unfermented forms such as tofu or soy milk but also fermented, such as tempeh, miso or natto. Soy nuts are produced from soybeans by soaking, draining, and then baking or roasting. Soy protein isolate is exceptionally high in protein and used widely across the food industry. Soy commonly substitutes for meat, most often as textured vegetable protein in meat alternative or vegetarian dishes. The nutritional and health benefits of soy and soy products have been recognized for some time, being ascribed to varying components of soybean and having a role in cardiovascular, bone and menopausal health [92,93]. A body of research on an association between soy and cognitive health and dementia risk is now accumulating. Within these studies, there have been distinct focuses on total soy consumption, soy protein content, bioactive peptides, isoflavones and fermented/non-fermented soy.

One of the earliest RCTs in this field failed to demonstrate evidence of an isoflavone containing soy protein supplement on any health parameter, including cognitive function, in healthy women who entered the study at the age of 60 years or later. However, concerns were raised at the time of publication about the methodology and dosage of the soy protein supplement, so the findings should be considered in light of these concerns [94]. A hypothesis some while later proposed that intake of soy and soy products could be a cause of AD and cautioned on reducing intake of this commodity. However, the authors were unable to support this with direct evidence, and no follow-up research has since appeared [95]. Early evidence of a positive relationship between soy and isoflavone intake and risk of cognitive impairment in elderly women was observed in a study in Japan in 2018. Cognitive function was assessed using the Mini-Mental State Examination (MMSE) and dietary intake by a 3-day diet record. Statistically significant findings suggested that total soybean and isoflavone consumption might decrease the risk of cognitive impairment in elderly Japanese women. This small-scale study would, however, be subject to risk of bias and statistical under-powering [96]. A 2018 cross-sectional study on Taiwanese adults aged ≥ 65 years explored the association between soy-based food intake and cognitive decline. It was found that soy-based foods were negatively associated with cognitive impairment. Although the study was observational in design, the authors suggested that the findings could be used as a point of reference for dietary recommendations in the elderly [97]. Following the findings of this study, a systematic review and meta-analysis focused specifically on soy isoflavones and cognitive function. Mean age of the participants from the 16 RCTs selected was 60 years. Overall, evidence supported the conclusion that soy isoflavones may improve cognitive function in adults and reduce the risk of cognitive decline and dementia, and soy isoflavones could be considered as a future intervention modality in this context [98]. Given the strengths of the review approach, high confidence can be placed in the conclusions and as a recommendation for supporting cognitive health in older individuals.

In contrast, an extensive prospective study of 18,991 men and 22,456 women was conducted in a Japanese population aged 45–74 years. Total soy, soy product and isoflavone intake were evaluated in relation to incident disabling dementia. A FFQ was used as the dietary assessment tool. On this occasion, the authors found no association between total soy product intake and disabling dementia, although the fermented product natto was marginally inversely associated in women. No association between any soy product or isoflavone intake and disabling dementia was found in men [99]. Several limitations to the study were highlighted, but the positive findings in relation to natto merit further investigation. Concerns about the sensitivity of the dietary assessment tool used could be raised in detecting accurate intakes, particularly in relation to isoflavone consumption.

Cerebral blood flow (CBF) is a marker of cerebrovascular function. This physiological parameter has been evaluated alongside psychomotor speed (a domain of cognitive function) in relation to longer-term consumption of soy nuts in older adults. Although a small-scale study, CBF and psychomotor speed were shown to increase in relation to longer-term soy nut consumption. These findings suggest a potentially beneficial effect of soy-rich foods on cognitive health in older adults and merit further examination using a more robust study design and larger sample size [100].

Several studies have included an exercise element when assessing the impact of soy consumption on dementia risk and memory function in older adults. A soy peptide product derived from an enzymatic hydrolysis of soy protein isolate has been evaluated in combination with exercise on memory function in community-dwelling older adults. This small-scale study demonstrated that after a 3-month intervention period, ingestion of the soy peptide formula resulted in a significant increase in memory score and cognitive function [101]. This study raises the potential for soy-protein isolate products to be used as a novel commercial concept to support cognitive health in older adults. However, assurances of the ability of the tools to adequately assess memory and cognition would be required to ensure validity of the study design.

Fermented soy possesses specific bioactive compounds not always present in the non-fermented form and, as such, may have different or enhanced impact upon health, including cognitive health [102]. Fermented soy powder has been evaluated in a RCT on cognition in a small group of adults aged ≥65 years over a 12-week period. A range of cognitive tests were administered. Those consuming fermented soy showed significant improvements in memory scores and global cognition. The authors concluded that although no causality can be inferred, fermented soy consumption had the potential to be a low-cost approach to delaying cognitive decline [103].

Soyasaponins, which include soyasapogenol, are a group of bioactive compounds found in fermented and unfermented soybeans and are being evaluated in relation to their biological and health-related properties [104]. Soyasapogenol B has been shown in an experimental mouse model to promote protein synthesis in skeletal muscle, suggesting a potential role for the soy-derived molecule in sarcopenia prevention [105]. However, whilst the pathway by which this is achieved has been identified, this early observation needs to be replicated and evaluated in human trials. Currently, Soyasapogenol B is being evaluated by in vitro studies as a potential agent against diseases such as AD [106].

Evidence on protein intake, sarcopenia and cognitive decline in the elderly has progressed with a randomized double-blind placebo-controlled study using a multinutrient-enriched soy flour (MNSF) (and soy flour as control) on body composition and cognitive function in elderly participants. Following a 12-week intervention period, the MNSF group showed significant improvements in skeletal muscle mass as well as other body composition parameters, and a significant decrease in the Mini-Mental State-Examination (MMSE) (used to measure cognitive impairment) [107]. Whilst the validity of this assessment tool may be an issue, the study design was robust and, nevertheless, a multinutrient-enriched food based upon soy could be a useful future addition to the supplement field to improve nutritional status, muscle health and delay cognitive decline in the elderly.

Soy as a source of choline is often overlooked and overshadowed by egg as the major dietary provider of this nutrient. To date, no soy-choline-specific studies have been conducted in the health field, but it is likely that, similar to egg, soy choline also contributes to brain structure and function and may well support cognitive health. Future studies should be able to support this assertion.

In conclusion, and on balance of the evidence, the consumption of soy and soy products in a dietary or supplemental form could potentially play a role in both delaying the onset of, and slowing down, the physical and cognitive decline of older adults through its provision of additional dietary protein as well as other specific bioactive compounds, including flavonoids [108]. More detailed and better designed studies should help to clarify where ambiguities may exist, and as for egg, molecular mode(s) of action should be explored in more depth.

### 7.3. Dairy

Protein-rich dairy food comprises a range of products including whole milk, whey protein isolate, yoghurt and cheese. Dairy consumption has been evaluated in relation to cognitive health either as an overall food group or as individual dairy foods. Whey protein has been evaluated in a range of health and exercise performance studies. Whey constitutes approximately 20% of milk proteins, together with lactose and micronutrients, being a soluble byproduct of cheese production. Further processing produces whey protein isolate, which has wide uses in the food industry, particularly the supplement and sports nutrition sectors [109]. The ANCHORS A-WHEY clinical trial evaluated daily (50 g) whey protein versus isoenergetic carbohydrate supplementation (for 12 weeks) in relation to a range of cardio- and cerebrovascular parameters and cognitive function in community-dwelling older adults. The study sample was statistically powered, and whilst the supplement led to favorable outcomes in cardiovascular measures, limited effects were observed on cognitive and cerebrovascular function. Study limitations, including a lack of control of dietary intake, were acknowledged and the authors concluded that interventions of a longer duration were warranted [110]. A later study of a similar duration evaluated the combination of resistance exercise and whey protein supplementation on cognitive function in older men (67 ± 4 years). On this occasion, improvements in executive function, with a trend towards improved global cognitive function, were found compared with the control group. The resistance exercise did not lead to any improvements in cognitive function. The study supported the role of dietary protein in healthy aging. However, the small sample size would indicate potential for an under-powered study [111]. A 2025 RCT evaluated a 12-month intervention with an enriched whey protein powder on cognitive function in older Chinese adults with mild cognitive impairment. Here, cognitive scores were improved in the whey protein group compared with those in the control group. As a secondary outcome, significant beneficial effects on skeletal muscle gain were found, suggesting a compensatory effect on muscle loss in aging. The authors recommended longer and larger RCTs to examine the effects of whey protein in brain structure and function [112].

Total dairy food consumption has also been evaluated on cognitive function in older adults. Using data on 7945 participants from the Canadian Longitudinal Study on Aging, cross-sectional associations between total and specific dairy product consumption (assessed using a FFQ) in relation to cognitive performance found that total dairy, cheese and low-fat dairy were positively associated with executive function domain, and yoghurt intake with memory domain. Despite the limitations associated with a cross-sectional study, the authors suggested that targeting dairy intake could benefit cognitive health-promoting interventions [113]. Data from the PREDIMED-Plus cohort, a randomized, parallel-group 6-year multicenter study, designed to explore lifestyle factors with cardiovascular disease risk, was used to assess the longitudinal (2 year) association between dairy intake with cognitive changes in a population of 4668 older Spanish individuals. They found a greater decline in cognitive function in those in the highest tertile of total milk and whole-fat milk consumption compared with those in the lowest tertile. No associations were found between low-fat milk, yoghurt, cheese or fermented dairy intake and cognitive performance [114]. These observations contrasted with those exploring dairy intake and incident dementia risk in a Japanese population of adults aged ≥ 65 years. Daily yoghurt consumers had a reduced risk, as did those consuming milk 1–2 times per month compared to non-consumers of milk. Cheese consumers, however, showed an increased risk. The authors could not confirm if the benefit from yoghurt consumption was specific to that dairy food or a marker of an overall healthy dietary pattern [115].

Fermented dairy foods, including yoghurt and cheese, have been suggested to have health-related properties not found in non-fermented dairy or non-dairy foods. As such, their consumption has been specifically evaluated in relation to cognitive function and dementia risk in older adults. Using data on older individuals in the NHANES 2011–2014 sample, Han et al. demonstrated that among yoghurt consumers (assessed through two 24 h recall interviews), global cognition was notably higher than in non-consumers, and concluded that in the elderly, moderate consumption of fermented dairy products is associated with better executive function and verbal fluency [116]. In an Australian study on adults aged 70–90 years, despite higher intakes of yoghurt and low-fat cheese being associated with lower risk of depression, cross-sectional and longitudinal analyses indicated no signification association with cognition or incident dementia [117]. The authors acknowledged several study limitations, including the possibility of recall bias due to a FFQ being employed to assess dietary intake. A 2026 prospective study examining the association between dairy intake and dementia risk in 27,670 participants showed that a higher intake of high-fat cheese and high-fat cream was associated with a lower risk of all-cause dementia. No significant association was observed for low-fat equivalent versions. Furthermore, APOE-ε4 status modified the association between high-fat cheese and AD risk [118]. Despite the many positive elements and strengths of the study, the authors recognized that potential confounding precluded the determination of causation and that diet was only assessed once at baseline.

As for egg and soy, evidence suggests that, on balance, dairy may be beneficial for cognitive health in older individuals, taking into account the stated limitations of the studies cited. Dairy also has the potential to enhance the protein content of the diet and help achieve intakes close to the recommended 1.0–1.2 g/kg/d for older adults. However, in such circumstances, attention should be paid to the fat content, as this could lead to overconsumption of energy and exceed energy requirements as well as increasing saturated fat intake. Low-fat options such as yoghurt and a drink-based whey protein isolate could be preferable in these situations.

In the context of protein supplementation and cognitive and physical health in older adults, BCAA supplementation has started to be evaluated [119]. BCAAs (isoleucine, leucine and valine) compete with other large neutral amino acids for carrier-mediated transport across the blood–brain barrier and entry into the central nervous system (CNS). Beyond limiting the transport of tryptophan into the CNS for the synthesis of serotonin and its role in central fatigue, a physiological rationale for BCAAs in cognitive health remains unclear. However well-designed intervention studies could provide better understanding of BCAA–cognitive health relationships in the elderly [120].

## 8. Functional Foods and Dietary Supplements for Cognitive and Physical Health in the Elderly

A shift has occurred in the health and medical sector towards greater emphasis on disease prevention and preventive nutrition. This has resulted in a rapid growth in the wellness industry, including the functional food and supplements sector. These products select foods and nutrients and package them into a format which is convenient, safe and palatable, often in the form of a drink or shake. The healthy aging sector targets a population group who tend to be motivated to maintain optimal health and function for as long as possible through diet and lifestyle. Equally, the frail elderly who are no longer able to live independently rely on care providers such as residential homes to ensure their nutritional and hydration needs are met. The translation of the evidence base into product development for functional foods for the aging population may be at a rudimentary stage, but there is potential to learn from current functional food products which meet or come close to the nutritional criteria to support aging-related physical and cognitive health (see later). However, functional foods should form part of a healthy varied diet and not be solely relied upon to maintain nutritional status and support functional wellbeing.

As the protein needs of the elderly may be greater than those of younger adults, intakes may not always be achieved even through recommended healthy eating patterns. This can especially be the case in individuals where appetite is poor and where eating ability might be limited. Two reviews have recently addressed the protein question in relation to drink-based nutrition in aged-care residents and the suitability of foods for protein fortification for older adults [121,122]. Protein-rich and nutrient-rich drinks contribute to the functional food market, either purchased or medically prescribed as a nutritional supplement for elderly individuals at risk of low dietary intake and therefore not meeting the potentially higher protein requirements. This type of functional food/drink has been experimentally assessed in physical and cognitive health studies. For example, a commercially available whey protein supplement/functional food has been evaluated in relation to muscle and metabolic parameters in diabetic sarcopenia. A number of positive outcomes were observed in the supplemented group, but caution should be exercised when interpreting the findings due to possible issues with sample size, duration of intervention and compliance [123]. A range of protein hydrolysates have been evaluated in relation to cognitive health in older individuals, and a docosahexaenoic-acid-enriched milk beverage has been shown to positively impact age-related cognitive decline [124,125]. Zajac et al. compared whey and soy protein isolates on cognitive function in older Australian adults, with the soy protein appearing to perform better at influencing cognitive tasks in older women compared with the whey equivalent. The authors acknowledged the limitation of duration of the intervention, although the sample size satisfied statistical power requirements [126].

The functional food market also encompasses a range of meal replacement products targeted at the healthy weight sector. Typically, they are defined as partial or total meal replacements with a low-energy formulation based around skimmed milk or whey protein, with plant versions also being available. Whilst some of these products have been evaluated in clinical trials focusing on weight loss as the primary outcome, few, if any, have been systematically tested outside the weight management field, such as in muscle mass gain and function. No study to date has evaluated a combination of soy and dairy in the context of sarcopenia and cognitive health. However, a formula comprising soy protein isolate, skimmed milk yoghurt and honey is used as a meal replacement for weight management [127]. The formulation has been extensively evaluated in a number of RCTs which have demonstrated optimal health and metabolic outcomes including weight loss, improvements in pre-diabetes status, metabolic health, body composition, skeletal muscle status, appetite and HRQoL [128]. This formulation might offer potential uses in the context of cognitive and physical (muscle) health in the elderly. Theoretically, elderly consumers could achieve an overall protein intake closer to the suggested 1.0–1.2 g/kg/d if consumed as a (low energy) supplement to a healthy varied diet. Incidentally, a third component of this functional food, honey, might also benefit cognitive health and dementia. Its flavonoid and phenolic compounds may exert neuroprotective and nootropic effects, justifying research to be undertaken in this area [129,130,131]. Thus, a closer examination of current or novel functional foods is warranted, particularly in a drink format, that could not only facilitate additional protein consumption of healthy older adults, but also specifically impact cognitive health and the delay of cognitive decline in this population group [132].

This review has purposefully focused on dietary protein and physical/cognitive health in older adults, but it must be acknowledged that other nutrients and dietary components have known or suggested benefits in these situations, including the contribution of the colonic microbiome [133]. This wider nutritional contribution has been addressed, in part, by the evaluation of whole-dietary patterns. However, the overall role of nutrition in cognitive health is beyond the scope of this review, and more general accounts are recommended [134,135].

## 9. Conclusions

Sarcopenia and cognitive decline are two non-communicable diseases associated with aging and an elderly population. This article has reviewed research which suggests that a link between these two conditions occurs with a further possible interaction with dietary protein intake. Aging is associated with “anabolic resistance” to protein synthesis in skeletal muscle. As protein requirement has been proposed to be higher in elderly individuals, this would need to be achieved via adjustments to typical healthy eating patterns to enhance the protein content. Evidence suggests that a higher protein intake might positively impact skeletal muscle and cognitive health in the elderly and possibly protect against cognitive decline. Established healthy dietary patterns such as the Mediterranean diet also have a role to play in preventive nutrition and cognitive health. It remains unclear whether any superiority exists between plant and animal protein in this situation. However, specific benefits from consumption of eggs, soy and dairy products on cognitive health have been identified. Protein-rich functional foods can play an important role in supporting the elderly to achieve their higher protein requirements. Further research could establish whether these protein-rich functional foods, particularly those which combine both soy and dairy proteins in the formulation, can help elderly individuals maintain muscle and cognitive health and add quality of life in older age.

## Data Availability

No new data were created or analyzed in this study. Data sharing is not applicable to this article.

## Abbreviations

AD	Alzheimer’s disease
ALM	Appendicular lean mass
APOE	Apolipoprotein E
ASM	Appendicular skeletal muscle
BCAA	Branched-chain amino acids
BIA	Bioelectrical impedance analysis
CBF	Cerebral blood flow
CLMLS	Chinese Longitudinal Healthy Longevity Survey
DASH	Dietary Action to Stop Hypertension
DIASS	Digestible Indispensable Amino Acid Score
DXA	Dual energy X-ray absorptiometry
EAA	Essential amino acid
FFQ	Food Frequency Questionnaire
HPFS	Health Professionals Follow-up Study
HRQoL	Health-related quality of life
MD	Mediterranean diet
MIND	Mediterranean-DASH Intervention for Neurodegenerative Delay
MMSE	Mini-Mental State Examination
MNSF	Multinutrient-enriched soy flour
NHANES	National Health And Nutrition Examination Survey
NHS	Nurses Health Study
RCT	Randomized controlled trial
SCD	Subjective cognitive decline
WHO	World Health Organization
